# Impact of Jilin Province Stroke Emergency Maps on Acute Stroke Care Improvement in Northeast China

**DOI:** 10.3389/fneur.2020.00734

**Published:** 2020-07-22

**Authors:** Hang Jin, Yang Qu, Zhen-Ni Guo, Xiu-Li Yan, Xin Sun, Yi Yang

**Affiliations:** ^1^Department of Neurology, Stroke Center, The First Hospital of Jilin University, Changchun, China; ^2^Department of Neurology, Clinical Trial and Research Center for Stroke, The First Hospital of Jilin University, Changchun, China

**Keywords:** acute stroke, thrombolysis, prehospital delay, ischemic stroke, stroke care

## Abstract

**Objectives:** Stroke burden is especially heavy in northeast China. Facilities with the capacity to perform acute reperfusion therapies for stroke are unevenly dispersed and are especially inadequate in rural areas. The aim of this study was to establish an effective measure to improve stroke emergency care, eventually increasing the capacity for reperfusion therapy in Jilin province, a less developed province in northeast China.

**Methods:** We created the Jilin province Stroke Emergency Maps (JSEM), a regional stroke emergency network. Qualified hospitals in Jilin province were integrated into JSEM according to strict inclusion criteria. With constant evaluation and screening, more qualified hospitals may be enrolled into the JSEM, which is updated and published once per year. Locations of hospitals with the capacity to perform intravenous thrombolysis and emergency mechanical thrombectomy were labeled on the map.

**Results:** After strict evaluation and screening, 19 designated hospitals were integrated into the JSEM in August 2017 (baseline). Following the implementation of the JSEM, 21 more designated hospitals (40 in all) were included in 2018, and 48 more designated hospitals were included in 2019. With the guidance of the JSEM, the rate of intravenous thrombolysis in Jilin province increased remarkably from 3.3% (2017, baseline) and 4.6% (2018) to 5.5% (2019). Mean door-to-needle time decreased from 62 min at baseline (2017) to 45 min 2 years later. The number of mechanical thrombectomy was increased from 457 at baseline (2017) to 749 (2018) and 1,137 (2019) per year, respectively, and mean door-to-puncture time was shortened from 136 to 120 min.

**Conclusion:** The JSEM, a regional stroke emergency network, serves to improve patient care for stroke. The map's publication increased rates of intravenous thrombolysis and mechanical thrombectomy. JSEM effectively connected more qualified designated hospitals, stroke patients and emergency medical service systems in Jilin province.

## Introduction

Effective treatments for acute ischemic stroke rely on timely restoration of blood flow for brain tissue. Currently, <3% of patients with ischemic stroke in China receive intravenous thrombolysis (1). The availability of hospitals with the capacity to perform acute reperfusion therapies for stroke patients is unevenly dispersed and is especially inadequate in rural areas (2). There is a population of nearly 27 million in the Jilin province, Northeast China, and a large portion of the populations is dispersed in rural areas that lack high-quality medical resources. It is assumed that the rate of stroke patients in Jilin province who receive emergency treatment within time window is much lower than average (3).

The Chinese government has instituted substantial strategies to reduce the burden of stroke. With the support of the Stroke Prevention Project Committee of the Chinese National Health Commission, The China Acute Stroke Care Mapping Steering Committee was established and Acute Stroke Care Maps have been implemented in more than 40 Chinese cities. Those maps integrate prehospital emergency medical service (EMS) systems and in-hospital strategies to provide improved pathways for Chinese acute ischemic stroke patients. In an effort to provide guidance to hospitals, EMS systems and residents in Jilin province, we established an effective measure, the Jilin province Stroke Emergency Maps (JSEM), the first province-wide stroke emergency map in China, to improve stroke emergency care. The purposes of this article are to present the designation, implementation, refinement, and improvement process of JSEM. We aim to eventually increase the capacity of reperfusion therapy after stroke, meet the needs of stroke patients, hospitals, and the EMS system in Jilin province.

## Materials and Methods

### Study Design

This is a retrospective observational study comparing the rates of intravenous thrombolysis and mechanical thrombectomy as well as EMS system response time in Jilin province using data 1 year before (Aug 2016–July 2017), 1 year after (Aug 2017–July 2018) and 2 years after (Aug 2018–July 2019) JSEM project initiation. The study was approved by the ethics committee of the First Hospital of Jilin University.

### Certification for JSEM

JSEM is a regional stroke emergency network. This project has been sponsored by the Jilin Province Health Ministry and implemented by the Jilin Stroke association and Jilin Stroke Care Quality Management Center since 2017. Jilin Stroke Committee of Experts was established to develop certification criteria of JSEM. The key components of qualified hospital for JSEM can be defined in 4 major areas revised and refined from recommendations for Primary Stroke Center (4) and Comprehensive Stroke Center (5, 6): (1) Qualification of hospitals; (2) Infrastructure; (3) Personnel with expertise; and (4) Quality control and improvement (Tables 1, 2). In addition, JSEM creatively developed two settings of certification criteria especially for screening hospitals qualified for intravenous thrombolysis and emergency mechanical thrombectomy according to 2018 AHA/ASA Guidelines (7). Qualified local hospitals that successfully met the certification criteria were awarded certification and integrated into JSEM. This strategy provided precise guidance for EMS systems to rapidly transfer patients with acute ischemic stroke to a properly qualified hospital.

**Table 1:** Intravenous thrombolysis qualification assessment criteria.

	**Evaluation elements**	**Score**	**Interpretations**
Qualification of hospitals (10)	Level of hospital, ability for IVT, stroke center certification	10	Grading tertiary hospitals scores 2; secondary hospitals scores 1.
			Primary stroke center scores 3 and comprehensive stroke center scores 5.
			Ability for IVT scores 3.
Infrastructure (25)	Equipped with equipment for ambulances communication	5	
	Pathway/procedures of stroke care	3	
	Emergency equipment including electrocardiogram, Multichannel telemetry; defibrillator, oxygen, alteplase/urokinase, first-aid medicine etc.	5	Deduct 1 point for each part missing.
	24 h/7 days Emergency CT	2	
	24 h/7 days Emergency laboratory test	2	
	Specific observation room for IVT	2	
	ED, stroke unit, rehabilitation department.	6	Deduct 1 point for each department missing.
Personnel with expertise (20)	24/7 Vascular neurologist	5	
	24/7 staff stroke nurse	5	
	24/7 neuroradiologist	5	
	Rehab-physicians or technicians	5	
Quality control and improvement (45)	Door to stroke team reception ≤ 15 min;	5	
	Door to CT scan initiation ≤ 30 min;	5	
	Door to laboratory result ≤ 45 min (Complete blood counts test, Blood biochemistry test, Rapid blood coagulation test).	5	
	DNT ≤ 60 min	10	
	IVT ≥ 30 cases per year	10	
	Clear written standard procedures protocol	5	
	Documentation and stroke care quality control	5	

*IVT, Intravenous Thrombolysis; CT, Computed Tomography; ED, Emergency Department; DNT, Door-to-Needle Time*.

**Table 2:** Mechanical thrombectomy qualification assessment criteria.

	**Evaluation elements**	**Score**	**Interpretations**
Infrastructure (30)	24 h/7 days CT angiography and perfusion scan	5	
	MRI, MRA	5	
	Emergency DSA	10	Deduct 1 point for each part missing.
	Neurointervention, Neurosurgery department and ICU.	10	
Personnel with expertise (30)	24 h/7 days operating room staffed	10	
	24 h/7 days vascular surgeon	10	
	24 h/7 days intensivist	10	
Quality control and improvement (40)	Stent/angioplasty	5	
	Hemicraniectomy	5	
	Reperfusion techniques (MT)	5	
	D2P ≤ 120 min	10	
	MT ≥ 10 cases per year	10	
	Clear written standard procedures protocol	5	
	Documentation and stroke care quality control	5	

*CT, Computed Tomography; MRI, Magnetic Resonance Imaging; MRA, Magnetic Resonance Angiography; DSA, Digital Subtraction Angiography; ICU, Intensive Care Unit; MT, Mechanical Thrombectomy; D2P, Door-to-Puncture*.

### Stroke Identification

EMS system staff identifies patients with suspected stroke according to “Stroke 1-2-0” (8) and recognizes patients for whom a suspicion of large vessel occlusion is strong by Rapid Arterial oCclusion Evaluation (RACE) scale ≥ 5 (9). Jilin Stroke Committee of Experts provided the training of these two prediction instruments and prehospital management of patients with acute ischemic stroke for EMS staff to ensure rapid and effective transport.

### Triage Paradigms of JSEM

**Figure 1:**
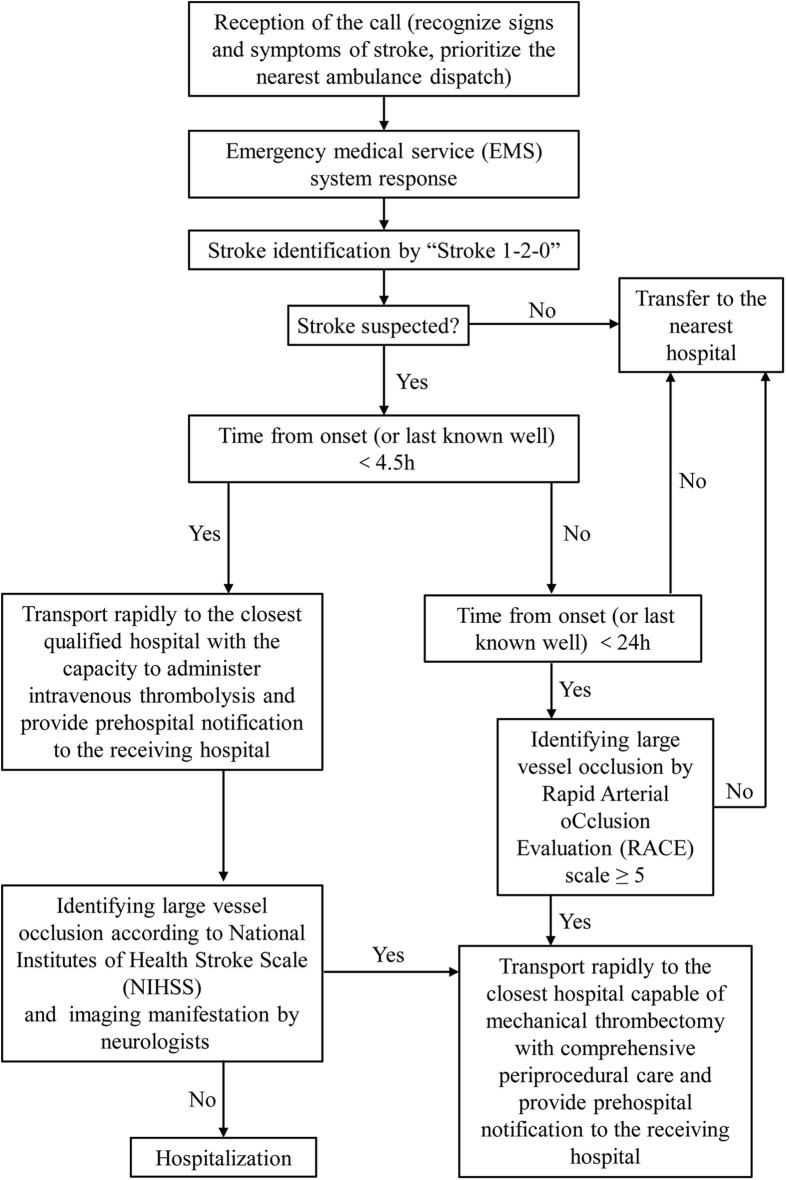
Jilin province stroke emergency maps (JSEM) transfer protocol.

Once the patient was suspected of stroke, EMS staff initiates operating procedures in accordance to JSEM transfer protocol (Figure 1). They should transport patients rapidly to the closest qualified hospital with the capacity to administer intravenous thrombolysis and provide prehospital notification to the receiving hospital to ensure earliest emergency department arrival. Quicker emergency department evaluation and rapid intravenous thrombolysis treatment to increase positive patient outcomes are needed. If the time from onset (or last known well) ≥ 4.5 h and the patient was under suspicion of large vessel occlusion, the patient should be transported rapidly to the closest hospital capable of endovascular stroke treatment with comprehensive periprocedural care. Meanwhile, EMS staff must consider the decision of patient and patient family members about which hospital they want to be transported to, avoiding unethical driving behaviors. Finally, neurologists in qualified hospitals decided the treatments according to the latest guidelines after communication with patient and patient family members.

### Training and Update for JSEM

Constant JSEM quality improvement initiatives are required. Regular trainings are provided by the committee of expertise of JSEM. These trainings ensure that the EMS staff recognizes stroke symptoms more precisely, the ambulances are dispatched more rapidly, emergency department staff react more efficiently, and stroke teams determine the appropriate therapeutic schedule more safely. Training programs are conducted every 3 months.

Jilin Stroke Care Quality Management Center is in charge of JSEM quality control. Multiple elements of stroke care quality benchmarks include the number of stroke patients who received intravenous thrombolysis or mechanical thrombectomy, mean door-to-needle time, mean door-to-puncture time, etc. All qualified hospitals are required to collect and submit their corresponding data quarterly. JSEM were updated and published once per year based on the quality control data.

## Results

### Implementation and Update of the JSEM

**Figure 2:**
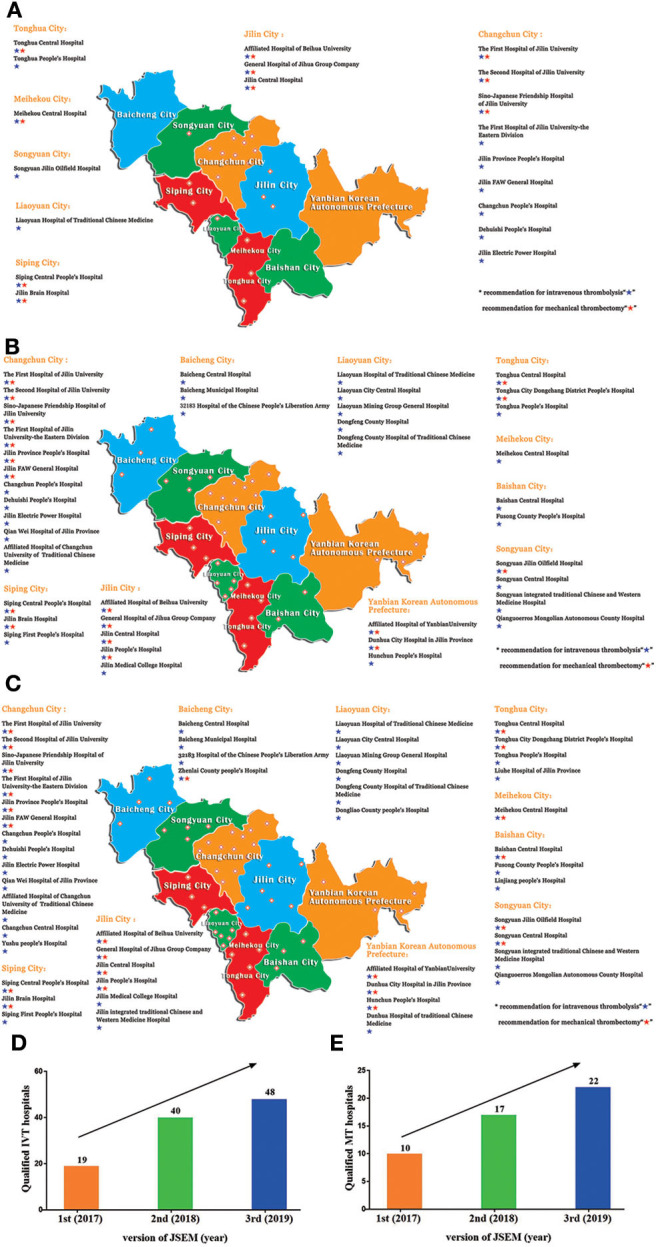
The **(A)** 1st, **(B)** 2nd, and **(C)** 3rd version of JSEM. The number of qualified **(D)** intravenous thrombolysis (IVT) and **(E)** mechanical thrombectomy (MT) hospitals was increased after the publication of JSEM.

Jilin province covers an area of 73,000 square miles and contains eight prefecture-level cities (Changchun, Baicheng, Baishan; Jilin City; Liaoyuan; Siping; Songyuan and Tonghua) and one autonomous prefecture (Yanbian). With strict evaluation and screening, the 1st version of JSEM was released in August 2017 (baseline). Nineteen designated hospitals capable for intravenous thrombolysis were integrated into the 1st map, among which only 10 hospitals are qualified for mechanical thrombectomy (Figures 2A,D,E). There were no qualified hospitals in two cities (Baishan, Baicheng) and Yanbian Korean autonomous prefecture on the 1st version of JSEM. Following 1 year of development, 21 more hospitals (40 in all) were designated as qualified to perform intravenous thrombolysis on July 2018. The number of hospitals capable of mechanical thrombectomy increased to 17 (Figures 2B,D,E). All the cities and prefectures were covered. In August 2019, 8 more hospitals were incorporated, and 22 hospitals were capable of performing mechanical thrombectomy (Figures 2C–E).

### Increasing Capacity of Intravenous Thrombolysis After JSEM Implementation

With the increasing number of designated hospitals in the JSEM capable of performing intravenous thrombolysis, the number of patients who received intravenous thrombolysis increased remarkably from 2,585 1 year (Aug 2016–July 2017) prior to JSEM project initiation, 3,834 per year (Aug 2017–July 2018), to 5,219 (Aug 2018–July 2019). The rate of intravenous thrombolysis increased correspondingly from 3.31% (2,585/78,063) at baseline JSEM, 4.59% (3,834/83,458) 1 year later to 5.5% (5,219/94,086) 2 years later. Mean door-to-needle time and mean onset-to-needle time was shortened from 62 and 238 min at baseline JSEM, 49 and 201 min after 1 year, to 45 and 196 min after 2 years, respectively (Table 3).

**Table 3:** Comparison of patients with acute ischemic stroke before and after implementation of JSEM.

	**Before**	**1 year after 1st version**	**1 year after 2nd version**
The number of patients received IV	2,585	3,834	5,219
Rate of IV thrombolysis	2,585/78,063 (3.3%)	3,834/83,458 (4.6%)	5,219/94,086 (5.5%)
Mean door-to-needle time (DNT) (min)	62	49	45
Mean onset-to-needle time (OTT) (min)	238	201	196
The number of patients received emergency MT	457	749	1,137
In patients received emergency MT, the ratio of bridging therapy	176/457 (38.5%)	277/749 (36.9%)	210/1,137 (18.5%)
Mean door-to-puncture (D2P) time (min)	136	127	120

*JSEM, the Jilin Province Stroke Emergency Maps; IV, Intravenous Thrombolysis; MT, Mechanical Thrombectomy*.

### Increasing Capacity of Mechanical Thrombectomy After JSEM Implementation

Due to the regular trainings provided by the JSEM committee, the hospitals met the mechanical thrombectomy qualification increased. Concomitantly, the number of patients who received emergency mechanical thrombectomy in Jilin province increased from 457 per year at baseline JSEM to 749 1 year later, 1,137 2 years later, and mean door-to-puncture time was reduced from 136 to 120 min. Among patients who received emergency mechanical thrombectomy, the ratio of bridging therapy was decreased year after year, from 38.5% (176/457) at baseline JSEM, 36.9% (277/749) 1 year later to 18.5% (210/1137) 2 years later (Table 3).

## Discussion

The JSEM was successful in increasing the number of qualified hospitals and patients who received intravenous thrombolysis and mechanical thrombectomy, and decreasing mean onset-to-needle, door-to-needle, and door-to-puncture times. More importantly, JSEM improved the overall acute stroke care level of Jilin province and strengthened the connection between hospitals, stroke patients, and EMS systems, which should be implemented in other provinces, especially less developed provinces in China, to aid in reducing stroke morbidity and mortality.

Stroke is one of the leading causes of death and disability at the national level in China. The National Epidemiological Survey of Stroke in China reported that the age-standardized stroke prevalence was 1,115 cases per 100,000 people, and mortality was 115 cases per 100,000. The highest incidence and mortality of stroke was in the Northeast region (365.2/100,000 person-years and 158.5/100,000 person-years, respectively) (10). Jilin province is located in Northeast China, which has borders with Russia and North Korea. It encompasses an area of 190,000 square km and the population is around 27 million. It is a less developed province in China; its age-standardized years of life lost (2,100 vs. 2,000 per 100,000 population) and disability-adjusted life-years (1.52 vs. 1.33 per 100,000 population) of stroke were significantly higher than the national averages (11).

### The Role and Benefit of JSEM

To the best of our knowledge, JSEM is the first province-wide stroke emergency map in China. Prior to establishment of the JSEM, several city-wide stroke emergency maps have been published in Shenzhen city (12), Shenyang city, and Qingdao city. These cities are densely populated and evenly distributed and located in developed areas with adequate medical resources. However, Jilin is a less developed province and most of the population is dispersed in rural areas that lack high-quality medical resources. If the map covered only Changchun city, the capital of Jilin province, most residents of our province would not benefit. JSEM demonstrated all the locations of qualified hospitals certified by the Jilin Province Health Ministry. We creatively labeled the recommendation for intravenous thrombolysis and emergency mechanical thrombectomy for each hospital on this map, which can assist hospitals and EMS system so that stroke patients in Jilin Province receive timely care that is most appropriate for their clinical condition. Moreover, after multifaceted quality improvement intervention organized by the JSEM committee, for example, sending doctors in rural areas to qualified hospitals for further study and providing regular guidance to unqualified hospitals, more and more hospitals met the intravenous thrombolysis and mechanical thrombectomy qualification and was integrated into JSEM. The baseline JSEM did not cover all the cities and prefecture, and regional distribution of qualified hospitals was extremely uneven, for instance, most are located in urban areas; however, more than two-thirds are located in the central part of Jilin province (Changchun, Jilin city, Liaoyuan, and Siping). On the contrary, there was no qualified hospital in Yanbian Korean autonomous prefecture, a mountainous country. Although sparsely populated (2.1 million), it makes up around one fourth of Jilin's land area. There may not be a stroke center located nearby for local patients in this region, which is inadequate for emergency stroke care. With the development of JSEM, there are now four identified qualified hospitals are capable of providing intravenous thrombolysis and three of them meet all of the requirements for emergency mechanical thrombectomy in Yanbian Korean autonomous prefecture 2 years later.

The implementation of JSEM was also successful according to the increased rate of intravenous thrombolysis and decreased onset-to-needle time, reflecting the transportation of patients is more effective. Traditional Chinese thought considers large scale comprehensive hospitals to be more reliable, thus, a large portion of acute ischemic stroke patients may choose a comprehensive hospital despite the long distance rather than the nearest one. As a result, they may miss the critical time window for optimal stroke treatment. Under the guidance of JSEM, traditional stereotypes are broken and more eligible patients with acute ischemic stroke can be transported to the nearest qualified hospitals within the appropriate time window. Door-to-needle time decreased, likely due to prehospital hospital notification and unobstructed in-hospital work-flow as a result of the regular trainings provided by the JSEM committee.

The total number of patients who received emergency mechanical thrombectomy increased rapidly, partly due to our JSEM with the recommendation of mechanical thrombectomy. Alternatively, this may also be due to the extension of the endovascular thrombectomy time window (13, 14). Specifically, among patients who received emergency mechanical thrombectomy, the ratio of bridging therapy was decreased each year, which indicates that the number of patients who received direct thrombectomy increased actually. Interestingly, number of patients receiving bridging therapy decreased 2 years later comparing with 1 year after JESM publication. In recent years, it is controversial whether acute ischemic stroke patients with large vessel occlusion who are eligible for both intravenous thrombolysis and mechanical thrombectomy benefit from pretreatment with alteplase or not. Interventional neurologists in hospitals with both intravenous thrombolysis and mechanical thrombectomy qualifications may choose direct mechanical thrombectomy other than bridging therapy in some conditions (for example, patients with atrial fibrillation or older patients) considering that thrombolysis prior to intra-arterial trombectomy might increase the incidence of symptomatic intracerebral hemorrhage, limit additional procedures during mechanical thrombectomy, such as administration of antiplatelet, cause thrombus fragility and thrombus migration and so on (15). This phenomenon is becoming increasingly common in China and may lead to the decrease in the number of bridge therapy. With the enhancement of acute ischemic stroke therapy education for the public and the increased number of recommended hospitals for emergency mechanical thrombectomy, the patients who receive direct thrombectomy is expected to further increase.

Overall, the success of the JSEM indicates that similar emergency maps should be implemented by other provinces, especially less developed provinces in China to further reduce stroke morbidity and mortality. The implementation of this emergency map not only helped to establish the prehospital emergency network to shorten transportation time and prompt the formation of stroke centers in some rural areas but also can improve awareness of stroke among the public to strengthen compliance with primary and secondary prevention methods.

### Limitation

There are also some limitations in this study. First, the increasing incidence of stroke in China year by year would also have an impact on the results, however, the rates of intravenous thrombolysis may partly overcome the influence of stroke incidence comparing with the number of intravenous thrombolysis. Mean door-to-needle time, mean onset-to-needle time and mean door-to-puncture may not be impacted by stroke incidence. Second, we did not carry out quality control assessments of the staff of EMS at present, so we do not have data for EMS and the accuracy of stroke identifications by EMS staff of JSEM could not be analyzed.

### Perspective

To further improve JSEM quality control, several initiatives are needed as follows: First, the number of patients transported by EMS did not increased thus far, possibly because public awareness of stroke symptoms and treatment remains poor in Jilin province. A study from three cities in China indicated that only 18.8% of the patients used the EMS (16). Therefore, public stroke education should emphasize the importance of the therapeutic time window. Second, in terms of EMS staff, one study demonstrated that nearly one-third of stroke cases were not identified by ambulance doctors (16). Previous studies demonstrate that the use of prehospital tools, such as the Face Arm Speech Time (FAST) test and Cincinnati Prehospital Stroke Scale (CPSS) to screen for stroke can improve stroke identification (17, 18). Furthermore, “stroke 1-2-0” are used widely in China (8). Alternatively, the newest guidelines call for large vessel occlusion identification ability (19). However, currently, no scale predicts large vessel occlusion with both high sensitivity and high specificity (19, 20). As a frequently validated large vessel occlusion prediction instrument, the sensitivities and specificities of the Cincinnati Prehospital Stroke Severity Scale (CPSSS) ≥ 2 for suspected stroke patients ranged from 56 to 58% and 77 to 85%, respectively (20, 21). Furthermore, CPSSS was user-friendlier than RACE scale. EMS staff, the most important part of prehospital procedures, could be provided with exhaustive “stroke 1-2-0,” FAST test, CPSS, and CPSSS training to improve stroke identification. Third, recently, telestroke has been proven to be one of the most successful applications of telemedicine (22). A systematic review and meta-analysis indicates that intravenous alteplase eligibility decision through telestroke networks is safe and effective within the 3-h time window (23). It seems that telestroke is suitable for Jilin province. Stroke centers in cities, such as Changchun can provide remote guidance to urban centers to ensure the best interests of stroke patients in remote areas are met. Further, telemedicine may help identify patients who are suspected with large vessel occlusion, while primarily admitted to only thrombolysis-capable hospitals (12). These will be the next direction in the informatization.

## Conclusion

In summary, this study described the key components of JSEM, the first province-wide stroke emergency map in China, including its certification, triage paradigms, and training and update. The publication of JSEM not only integrated more intravenous thrombolysis and mechanical thrombectomy qualified hospitals, but also significantly increased the rates of intravenous thrombolysis and mechanical thrombectomy and decreased onset-to-needle, door-to-needle, and door-to-puncture times in a less developed province of China.

## Data Availability Statement

The raw data supporting the conclusions of this article will be made available by the authors, without undue reservation.

## Ethics Statement

The studies involving human participants were reviewed and approved by the Human and Research Ethics committees of the First Hospital of Jilin University. The patients/participants provided their written informed consent to participate in this study.

## Author Contributions

YY and Z-NG devised the study design and supervised study procedures. HJ, YQ, and X-LY analyzed the data and wrote the manuscript. XS collected the data. All authors provided critical review, edits, and approval of the final manuscript.

## Conflict of Interest

The authors declare that the research was conducted in the absence of any commercial or financial relationships that could be construed as a potential conflict of interest.
